# Research on emergency scheduling based on improved genetic algorithm in harvester failure scenarios

**DOI:** 10.3389/fpls.2024.1413595

**Published:** 2024-06-21

**Authors:** Huanyu Liu, Lihan Zhang, Baidong Zhao, Jiacheng Tang, Jiahao Luo, Shuang Wang

**Affiliations:** ^1^ Institute of Modern Agricultural Equipment, Xihua University, Chengdu, Sichuan, China; ^2^ School of Mechanical Engineering and Automation, Dalian Polytechnic University, Dalian, Liaoning, China

**Keywords:** harvester emergency scheduling, hybrid optimization algorithm, scheduling recovery strategy, scheduling timeliness, scheduling system

## Abstract

In response to the issue of harvesting machine failures affecting crop harvesting timing, this study develops an emergency scheduling model and proposes a hybrid optimization algorithm that combines a genetic algorithm and an ant colony algorithm. By enhancing the genetic algorithm’s crossover and mutation methods and incorporating the ant colony algorithm, the proposed algorithm can prevent local optima, thus minimizing disruptions to the overall scheduling plan. Field data from Deyang, Sichuan Province, were utilized, and simulations on various harvesting machines experiencing random faults were conducted. Results indicated that the improved genetic algorithm reduced the optimal comprehensive scheduling cost during random fault occurrences by 47.49%, 19.60%, and 32.45% compared to the basic genetic algorithm and by 34.70%, 14.80%, and 24.40% compared to the ant colony algorithm. The improved algorithm showcases robust global optimization capabilities, high stability, and rapid convergence, offering effective emergency scheduling solutions in case of harvesting machine failures. Furthermore, a visual management system for agricultural machinery scheduling was developed to provide software support for optimizing agricultural machinery scheduling.

## 1 Introduction

Wheat harvesting is highly time-sensitive, with the optimal period for harvesting being very short. As the saying goes, “Harvest at ninety percent ripeness for full yield; at full ripeness, ten percent is lost.” Therefore, the scheduling of agricultural machinery is a crucial component of modern agriculture and is closely related to the productivity of agricultural operations (Bochtis et al., 2014). Proper scheduling can strategically organize the operational time and areas for machinery, preventing aimless movement, reducing operational conflicts, and minimizing repeated operations. This optimization substantially enhances agricultural production efficiency, fostering the modernization and sustainable development of agricultural operations. It is evident that the advancement of modernized agriculture relies heavily on effective machinery scheduling processes (Chen et al., 2021; Li, 2022). However, the implementation of scheduling plans is inherently dynamic. During peak seasons, the high demand for harvesters and prolonged continuous operation can lead to inevitable machinery failures. Solely relying on manual emergency scheduling methods based on experience proves insufficient for addressing the complexities of real-life situations. The absence of a proficient emergency scheduling strategy can result in extensive delays in harvests, with crops missing their optimal harvesting periods and consequently delaying the progress of harvesting operations. Adverse effects ripple throughout the entire production chain (Liu D. et al., 2022; Brewer et al., 2022). Therefore, it is urgent to explore advanced emergency scheduling methodologies for harvesters in the event of failures to enhance the overall level of scheduling efficiency (Sun et al., 2022).

Currently, the agricultural machinery scheduling has received much attention and achieved fruitful results. Scholars have tackled the scheduling problem with intelligent algorithms (Zhang W. et al., 2022; Hou et al., 2022), such as genetic algorithms (Ma et al., 2022; Liu et al., 2024), ant colony algorithms (Wang et al., 2023), particle swarm algorithms (Worasan et al., 2020; Huang et al., 2023), and hybrid optimization algorithms (He et al., 2019; He and Li, 2021; Feng et al., 2024). Similar intelligent algorithms have improved computational efficiency and the ability to make fast and accurate decisions in scheduling agricultural machines, providing a powerful tool for realizing rational agricultural machinery scheduling (Zhang W. P. et al., 2022; Zhang F. et al., 2022). In static scheduling research, various scholars have explored different perspectives, presenting a layered progression of logical relationships. Plessen (2019) developed a harvest planning method based on the coupling of crop assignment and vehicle routing, addressing the optimal sequence for servicing fields of the same crop during harvest. He et al. (2018) proposed a wheat harvest scheduling model for agricultural machinery cooperatives in China, aiming to minimize the harvesting period on fragmental farmlands while considering the constraint of minimizing differences in harvesting times among different combine harvesters. Building on this, Wang and Huang (2022) extended the research by proposing a mixed-integer linear programming model that integrates operator assignment, aiming to minimize total working time and costs. Yang et al. (2022) introduced an improved whale optimization algorithm to optimize the scheduling of multiple types of combined harvesters, aiming to comprehensively minimize total costs. Li (2022) presented an intelligent scheduling method based on non-dominated sorting genetic algorithm III (NSGA-III) and an improved ant colony algorithm for multi-machine collaborative operations, aiming to reduce operation time and enhance efficiency. Tian et al. (2023) developed and validated a multi-objective waypoint planning algorithm for drones in orchard spraying, using an improved ant colony algorithm that optimizes nodes through an improved heuristic function and introduces a ranking optimization mechanism to accelerate algorithm iterations. Overall, these studies demonstrate the progressive evolution from basic scheduling models to intelligent optimization algorithms that improve the scheduling efficiency and operational effectiveness of agricultural machinery by comprehensively considering machines, operators, and operational environments.

The aforementioned study has made significant advances in static scheduling; however, it faces challenges in ensuring reasonable scheduling when encountering disruptive events. Agricultural machinery scheduling is a dynamic and complex process, and the emergence of agricultural machinery failures hinders the original scheduling plan, which needs to be changed for the first time. Some scholars have included dynamic factors in the study of agriculture machinery scheduling. For example, Okulewicz and Mańdziuk (2017) simplified dynamic vehicle scheduling by introducing a swift, event-responsive tactic. Cao et al. (2021) optimized task assignments by balancing the workload among agricultural machinery. Seyyedhasani and Dvorak (2018) applied enhanced heuristic algorithms for agricultural machinery scheduling, accommodating field-level dynamics. Hu et al. (2020) proposed a two-stage method for agricultural scheduling planning, validated through case studies. Liu et al. (2021) introduced an improved immune algorithm, enhancing cross-regional machinery scheduling efficiency. Furthermore, Fernandez et al. (2020) employed particle swarm optimization and shuffled frog-leaping algorithms to develop a dynamic charging scheduling scheme, optimizing charging costs and improving the economic efficiency of charging stations. Liu Y. X. et al. (2022) implemented a novel hyper-heuristic algorithm for multi-line bus dynamic scheduling, significantly reducing waiting times. In the realm of agricultural drone scheduling, Chen et al. (2023) introduced a Levy annealing algorithm, demonstrating exceptional performance in schedule optimization. Moreover, Fatemi-Anaraki et al. (2023) explored the impacts of rescheduling within robotic manufacturing, providing insights into dynamic scheduling strategies. Taken together, these investigations signal an evolution toward advanced algorithmic optimization, which is critical for enhancing the dynamic scheduling of agricultural machinery and operational efficiency in farming practices.

However, the aforementioned literature has not yet conducted an in-depth study on harvester malfunctions, and the solutions for other disruptive events mostly involve complete rescheduling for optimization. These methods can cause significant disruption to the entire system, especially in the highly time-sensitive field of agriculture. This paper conducts an in-depth study on harvester malfunctions, aiming to address the disruptions caused by such failures and minimize the resulting losses. This study utilizes genetic algorithms (GA) as its primary framework, enhancing crossover and mutation techniques while incorporating ant colony algorithms to boost the overall algorithm’s local search capabilities. The objective is to enable prompt and precise decision-making in the face of harvester failures, ultimately minimizing the impact of breakdowns on scheduling plans. The key contributions of this study include the following:

(1) Developed an emergency scheduling model focused on minimizing the overall scheduling cost, taking interference management as the central concept and utilizing the theory of multi-agricultural machinery scheduling operations as the foundation. The objective is to mitigate the disruption to the scheduling plan in case of a harvester breakdown.
(2) Designed the solution algorithm of the model. The genetic algorithm was used as the framework, and the crossover and mutation methods were improved. The ant colony algorithm was introduced to improve the local search ability of the overall algorithm to reduce the impact of the scheduling plan due to harvester failure.
(3) In the case study, the improved genetic algorithm proved to be superior in emergency scheduling in case of harvester failure by constructing a visualized management system for scheduling agricultural machinery and selecting actual data for simulation tests.

## 2 Agricultural emergency scheduling models

### 2.1 Problem description

**Figure 1:**
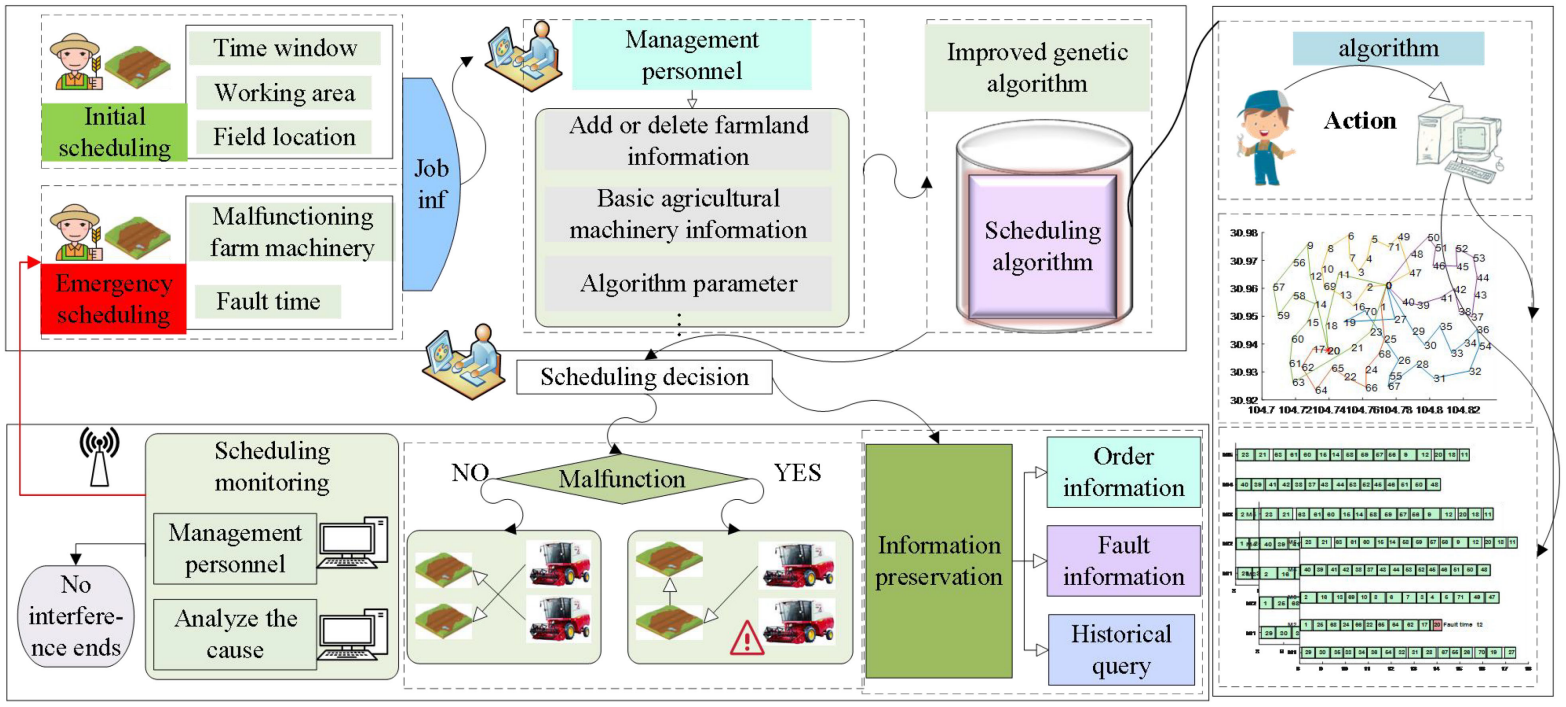
Schematic diagram of the scheduling process for agricultural machinery.

The emergency scheduling problem in case of a harvester breakdown can be described as follows: an agricultural cooperative schedules *M* = {*M*_1_, *M*_2_,…, *M_M_*} harvesters to *F* = {*F*_1_, *F*_2_,…, *F_N_*} farmland for operation, and when *k* harvesters break down and are unable to continue their operational tasks, the remaining *M* = {*M*_1_, *M*_2_,…, *M_M_*_−_ *_k_*} harvesters operate on the remaining farmland according to the scheduling goal and return to the agricultural cooperative after completing the operation. The whole scheduling process is shown in **Figure 1**.

Prior to the study, hypothetical preconditions for this emergency scheduling problem are first presented:

(1) Each farmland requires only one harvester to operate.
(2) All harvesters are traveling at a uniform speed during the transfer process.
(3) Broken-down harvesters return to the farm cooperative for repair by default.
(4) All functioning machinery returns to the machinery depot after completing their tasks.
(5) Only faulty harvesters break down into disturbing events during the scheduling process.
(6) All harvester operations progress according to the scheduling plan.
(7) It is assumed that all harvesters in the farm cooperative have been tasked and that no additional assignments will be considered.
(8) The location information of the farmland and harvesters is known, and the time window of the farmland is known and does not change.
(9) In this research study, a soft time window constraint is used, which will incur waiting costs if the harvester is earlier than the operating time and penalty costs if the operating time is exceeded.

### 2.2 Mathematical model

Based on the problem description of emergency scheduling, the various influencing factors were measured, and the emergency scheduling model with a soft time window and the optimization objective of minimizing the integrated scheduling cost was developed. The set of farmland *F* = {*F*_1_, *F*_2_,…, *F_N_*}, with *F_i_* representing the *i*th farmland, and its attributes are described as *F_i_* = {*Loc_F_i_*, *S_F_i_*}, where *Loc_F_i_* and *S_F_i_* denote the location and area of the farmland *F_i_*, respectively, and *i* ∈ [1, *N*]. The set of harvesters *M* = {*M*_1_, *M*_2_, …, *M_M_*}, with *M_k_* representing the *k*th harvester, is described by its attributes as *M_k_* = {*Loc_M_k_*, *V_M_k_*, *E_M_k_*}, where *Loc_M_k_* denotes the current position of harvester *M_k_*, *V_M_k_* denotes the average traveling speed of harvester *M_k_* during the plot transfer process, and *E_M_k_* denotes the average operating speed of harvester machinery *M_k_*, *k* ∈ [1, *M*]. For the convenience of the study, the following mathematical symbols are defined, as shown in **Table 1**.

**Table 1:** Symbols and meaning.

Symbol	Meaning
*y*	Integrated scheduling costs
*S_i_*	Farmland area
*V*	Traveling speed
*k’*	Faulty harvester number
*t_ij_*	Node *i* to node *j* traveling time
*C_j_*	Operating hours for farmland *j*
*d_ij_*	Distance from node *i* to node *j* (Euclidean distance)
*P_k_*	Agricultural machinery *k* penalty cost
*Q_k_*	The maximum operation of agricultural machinery *k*
*D_k_*	The maximum driving distance of agricultural machinery *k*
*x_ijk_*	Agricultural machinery *k* travels from *i* to *j*
*x_ik_*	Agricultural machinery *k* servicing farmland *i*
*w_kj_*	Waiting time incurred by agriculture machinery *k* earlier than the earliest operating time at farm site *j*
*C_1_, C_2_, C_5_*	Unit transfer cost, unit operation cost, unit delay cost (CNY)
*C_3_, C_4_*	The unit time penalty cost C_3_ for early arrival and C_4_ for late arrival (CNY)
*t′_ki_*	The time in the emergency scheduling plan when agriculture machinery *k* starts servicing farmland *i*
*t_ki_*	The time in the initial scheduling plan when agriculture machinery *k* starts serving farmland *i*
*t_kj_*	The time in the initial scheduling plan when agriculture machinery *k* starts serving farmland *j*
*Ei*	The earliest working time of node *i*
*Li*	The latest working time of node *i*

Objective function:


(1) $$\min y=C_{1}\sum_{k=1}^{M}\sum_{i=1}^{N}\sum_{j=1}^{N}x_{ijk}d_{ij}+C_{2}\sum_{k=1}^{M}\sum_{i=1}^{N}x_{ik}s_{i}+\sum_{k=1}^{M}P_{k}+C_{5}\sum_{k=1}^{M}\sum_{i=1}^{N}x_{ik}\left|{t^{\prime }}_{ki}-t_{ki}\right|$$

Constraints:


(2) $$\sum_{i=1}^{N}x_{ik}=1;\;(k\neq k^{\prime })\;$$

(3) $$\sum_{i=1}^{N}x_{ik^{\prime }}=0\;;$$

(4) $$\sum_{i=1}^{N}\sum_{j=1}^{N}x_{ijk}S_{i}\leq Q_{k};(k\in M)$$

(5) $$\sum_{k=1}^{M}\sum_{i=1}^{N}x_{ijk}=1;(j\in N)$$

(6) $$\sum_{i=1}^{N}\sum_{j=1}^{N}x_{ijk}d_{ij}\leq D_{k};(k\in M)$$

(7) $$P_{k}=\left\{ \begin{array}{l} x_{ik}(E_{i}-t_{ki})C_{3}\;\;\;\;\;t_{ki}<E_{i}\\ \;\;\;\;\;\;\;\;\;\;0\;\;\;\;\;\;\;\;\;\;\;\;\;\;\;\;E_{i}\leq t_{ki}\leq L_{i}\\ x_{ik}(t_{ki}-L_{i})C_{4}\;\;\;\;t_{ki}>L_{i} \end{array}\right.\;\;;(i\in N)$$

(8) $$w_{ki}=max(0,Ei-t_{ki});(i\in N,k\in M)$$

(9) $$t_{ij}=\frac{d_{ij}}{V};(i,j\in N)$$

(10) $$t_{ki}=t_{kj}+w_{kj}+c_{j}+t_{ij}$$

(11) $$x_{ijk}=\left\{ \begin{array}{l} 1,\;\;\text{Harvester }k\text{ travelling from }i\text{ to }j\\ 0,\;\;\text{other} \end{array}\right.$$

(12) $$x_{ik}=\left\{ \begin{array}{l} 1,\;\;\text{Harvester }k\text{ operating on farmland}\;i\\ 0,\;\;\text{other} \end{array}\right.$$

The objective function Equation 1 represents the comprehensive scheduling cost minimization as the objective function, where the first part is the objective function for the initial scheduling, which is the sum of the three of the scheduling total traveling cost, job cost, and penalty time cost, and the second part is the offset cost after the breakdown occurs; the final objective is to minimize the comprehensive scheduling cost as the goal. Equation 2 ensures that, in the event of a failure, operational harvesters are scheduled from their current locations. Equation 3 ensures that faulty harvesters are not scheduled. Equation 4 indicates that the operating capacity of each piece of agriculture machinery cannot exceed its maximum operating capacity limit. Equation 5 indicates that each piece of agriculture machinery operates only once in each field. Equation 6 indicates that each piece of agriculture machinery does not operate beyond its maximum distribution distance. Equation 7 represents the penalty cost constraint, where the agriculture machinery arrives at the field earlier than the earliest operation time, incurring a waiting cost, and later than the latest operation time, incurring a penalty cost. Equation 8 represents the waiting time constraint. Equation 9 represents the traveling time constraint. Equation 10 represents the time constraint that the current time to reach the farm field = time to reach the previous field + waiting time + previous farm operation time + travelling time. Equation 11 represents the decision variable: the decision variable of whether or not agriculture machinery *k* travels from farm field *i* to farm field *j*. Equation 12 represents another decision variable: the decision variable of whether agriculture machinery *k* serves in farm field *i* or not.

## 3 Design of the improved genetic algorithm

### 3.1 Analysis of model-solving algorithms

**Figure 2:**
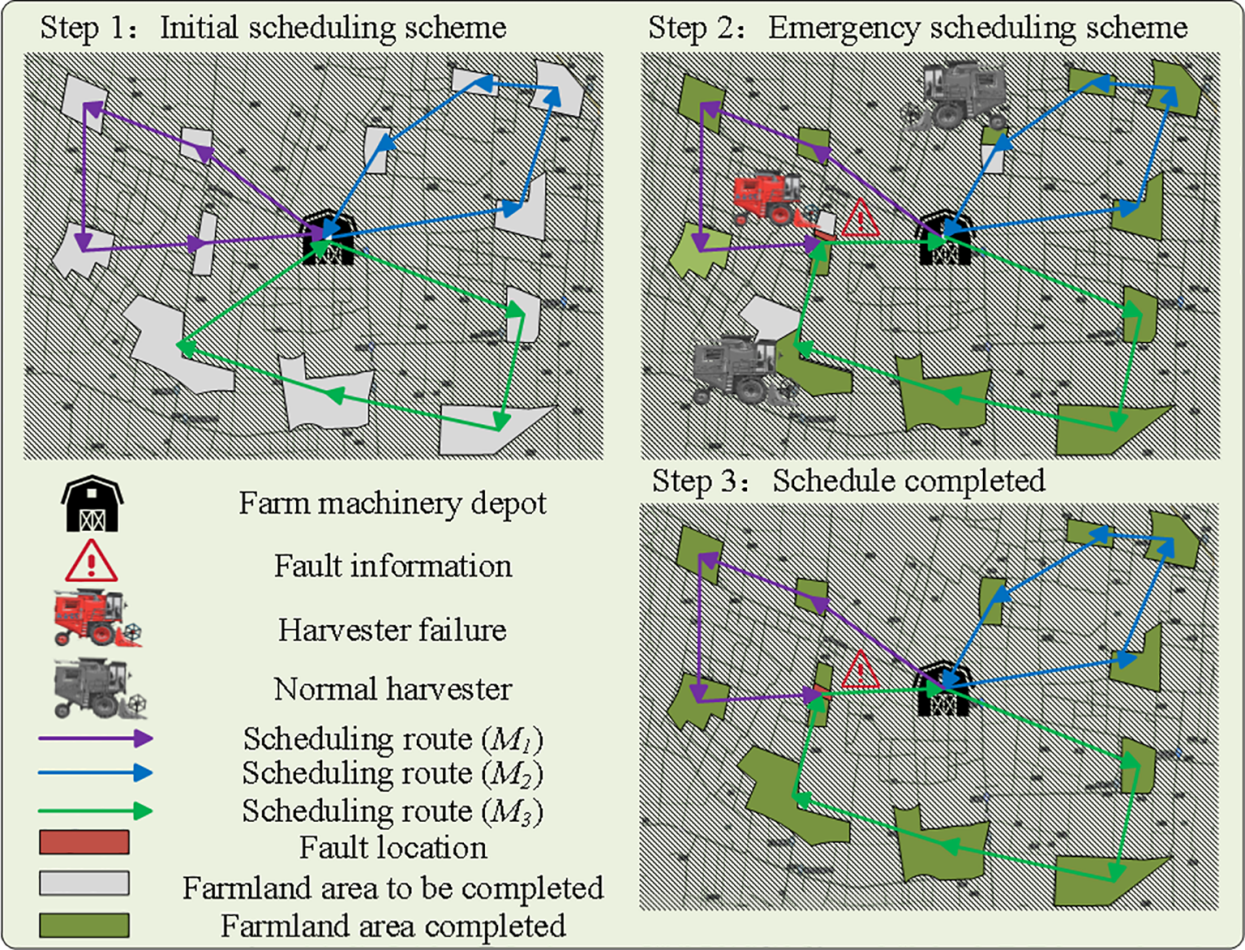
Schematic diagram of the emergency scheduling process.

By analyzing the agricultural machinery scheduling problem, it can be approached and solved similarly to the emergency vehicle scheduling problem. The solution process for emergency scheduling is illustrated in **Figure 2**.

**Figure 3:**
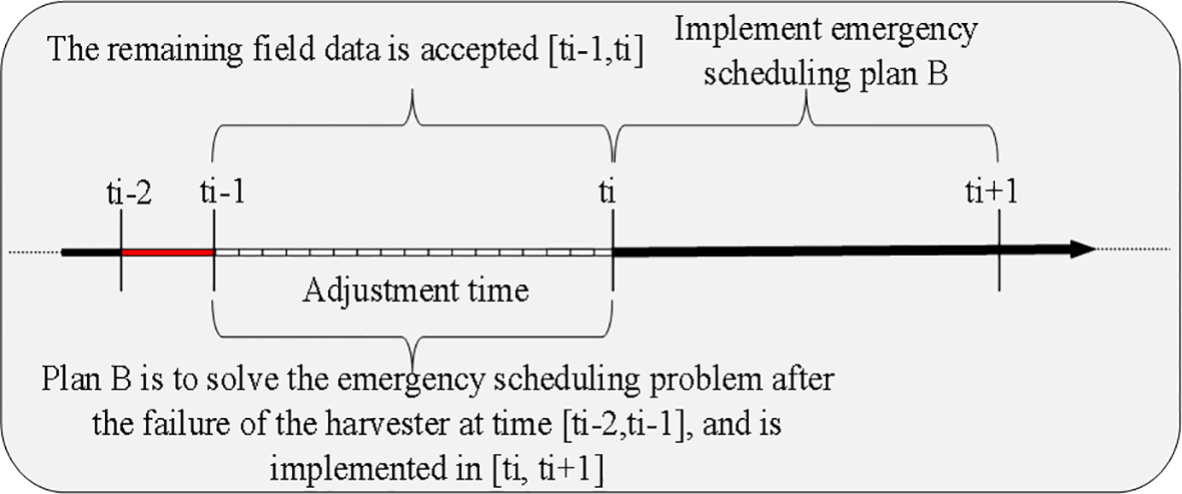
Emergency scheduling process schematic.

Upon receiving a breakdown scheduling request at the information center of the agricultural cooperative, the breakdown request needs to be processed immediately. The optimization objective is to minimize the comprehensive scheduling cost while ensuring that the remaining operating capacity of the agricultural machine in the farmland, the distance, and the offset time are within feasible limits. An emergency scheduling plan should be quickly incorporated into the operation task rather than completely re-scheduled. The process of receiving and scheduling the remaining farmland after the breakdown of a piece of agriculture machinery is illustrated in **Figure 3**.

### 3.2 Selection and design of model-solving algorithms

#### 3.2.1 Choice of model-solving algorithms

To solve the emergency scheduling problem in harvester breakdown scenarios, a heuristic algorithm is considered the optimal choice for addressing dynamic problems owing to its capacity to effectively solve challenges through an evolutionary process. Therefore, a genetic algorithm combined with an ant colony algorithm (GA-ACO) was proposed, which is based on the GA framework and introduces ACO to further add a local optimal approach. First, GA, as a typical genetic optimal framework, achieves global optimality in solving various scheduling problems. Second, ACO preserves the pheromone information in the environment in advance, which is very useful when there is a small change in the environment, and constructs the whole scheduling scheme directly by adding the non-working farmland to the current partial scheduling scheme. The combination of the two algorithms not only complements their respective drawbacks but also takes into account the difficulties of scheduling complexity to obtain a better contingency scheduling solution.

#### 3.2.2 Design of model-solving algorithms

**Figure 4:**
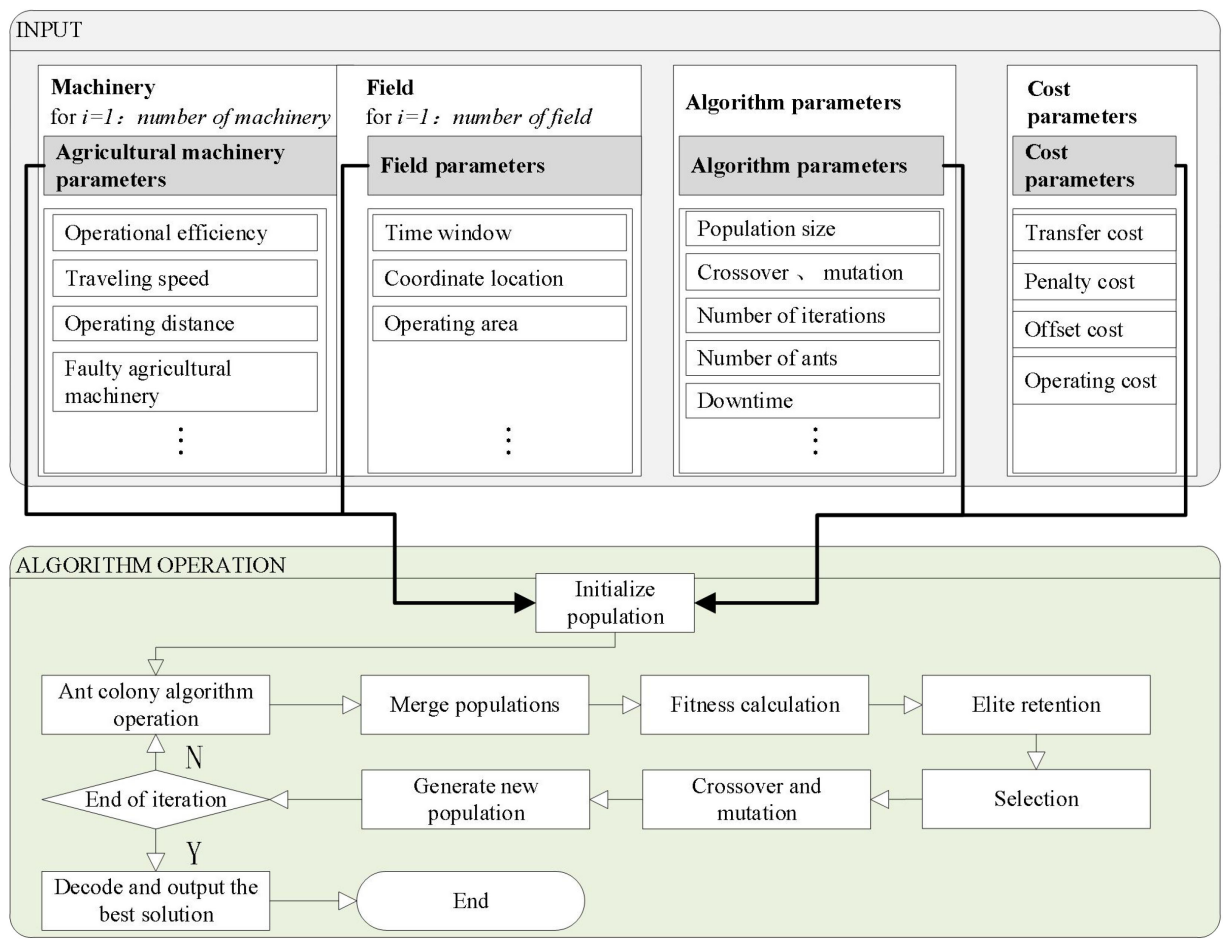
Improved genetic algorithm flowchart.

The flowchart of the algorithm is shown in **Figure 4**. The specific design steps of the improved genetic algorithm are as follows:

(1) Input the number of the faulty harvester, the moment of breakdown, and the parameters of the agriculture machinery; get the farmland to be operated with the normal harvester; and finally, add it to the emergency scheduling sequence. The specific steps are as follows **Algorithm 1**.

**Algorithm 1:** Emergency scheduling information extraction.

**Figure 5:**
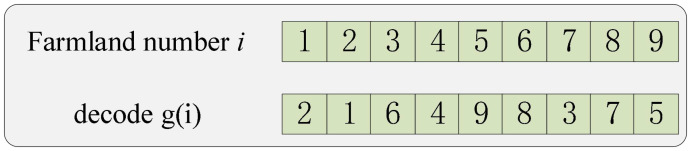
Decoding process.

**Input**: V_Fault %Fault harvester; T_Fault_Time %Fault time; Initial_V %Number of original harvesters; Initial_Route %Original scheduling route Initial_Time %Original scheduling time **Output**: Farmland, V %harvester 1: Farmland=[]; 2: V=Initial_V; 3: V_Fault=0 4: **while** Extraction of farmland to be operated **do**: 5: **if** V= V_Fault **then** 6: **if** breakdown in the field **then** 7: a=Find (Initial_Time > =T_Fault_Time) 8: V_Fault = V_Fault+1 9: **else** 10: a=Find (Initial_Time > T_Fault_Time) 11: V_Fault = V_Fault+1 12: **else if** 13: **else** 14: a=Find (Initial_Time > T_Fault_Time) 15: **end if** 16: update Farmland=Initial_Route{a}; 17: **end while** 18: V=V-V_Fault;

(2) The algorithm uses integer coding for encoding and decoding. The chromosome length corresponds to the number of operating farmlands (1 to *n*). The initial population is generated randomly. The algorithm prioritizes operations based on the distance between farmlands and agricultural machinery, as well as operation time. It then assigns operation points to each piece of agricultural machinery based on these priorities. As shown in **Figure 5**, where the number of farmlands is 9, *g*(*i*) denotes the priority of operation point *i*, where the smaller number of *g*(*i*) means the higher priority of the operation point. The order of operation of agriculture machinery obtained after decoding is 2–1-7–4-9–3-8–6-5.

(3) Improvement of the ant colony algorithm operation. The ACO is derived from the behavior of ants in searching for food, where ants mark paths by releasing pheromones and other ants choose paths with more pheromone concentration. In this study, the transfer probability in the ACO algorithm’s optimization strategy is shown in Equation 13, and the pheromone concentration is updated in Equations 14, 15. The solved populations are then merged into the genetic algorithm population for iteration.


(13) $$p_{ij}^{k}(t)=\left\{ \begin{array}{l} \frac{{\left[\tau_{ij}(t)\right]}^{\alpha }{\left[\eta_{ij}(t)\right]}^{\beta }}{\sum_{j\in allowed}{\left[\tau_{ij}(t)\right]}^{\alpha }{\left[\eta_{ij}(t)\right]}^{\beta }}\;\;,\;j\in allowed\;\\ \;\;\;\;\;\;\;\;\;\;\;\;\;\;\;\;\;0\;\;\;\;\;\;\;\;\;\;\;\;\;\;\;\;\;\;\;,\;\;\;\;otherwise\; \end{array}\right.$$

(14) $$\tau_{ij}=(1-p)\cdot \tau_{ij}+\sum_{k=1}^{m}\Delta \tau_{ij}^{k}$$

(15) $$\Delta \tau_{ij}^{k}=\left\{ \begin{array}{l} \frac{1}{d_{ij}},(i,j)\in T^{k}\\ \;0\;\;,otherwise \end{array}\right.$$

where *P^kij^* (*t*) is the probability that the *k*th ant chooses path *i* to *j*. α and β are parameters that adjust for the effects of pheromone concentration and heuristic information on path selection. *η_ij_* (*t*) is the heuristic information on path *i* → *j*, *τ_ij_* is the pheromone concentration of *i* → *j*, *ρ* ∈ (0, 1) is the volatility coefficient of the pheromone, *m* is the number of ants, Δ*τ^k^ij* is the pheromone left by the *k*th ant on path *i* → *j*, and *d_ij_* is the distance from node *i* to node *j*.

To prevent the algorithm from prematurely converging to a local optimum solution, we limit the pheromone concentration of each path to a predefined range (Equation 16), avoiding the “infinite loop” phenomenon.


(16) $$\tau_{ij}\left\{ \begin{array}{l} \tau_{ij}\;\;\;,\;\;\tau_{min}<\tau_{ij}<\tau_{max}\\ \tau_{min},\;\;\tau_{ij}\leq \tau_{min}\\ \tau_{max},\;\;\tau_{max}\leq \tau_{ij} \end{array}\right.$$

where *τ_max_* and *τ_min_* are the maximum and minimum pheromone settings, respectively.

We introduced a methodology (Liu Y. Y. et al., 2022) to enhance the efficiency of the update rule and then utilized a binary approach for optimization. Ants were ranked based on their traversal times across all regions after each iteration, with only the pheromones released by the top 50% fastest ants being retained, as described in Equations 17, 18.


(17) $$\tau_{ij}(t+1)=(1-p)\tau_{ij}(t)+\sum_{k=1}^{k\frac{m}{2}}[\sum_{k=1}^{w-1}\left(w-k)\Delta \tau_{ij}^{k}(t)]+e\Delta \tau_{ij}^{bs}(t\right)$$

(18) $$\Delta \tau_{ij}^{bs}(t)=\left\{ \begin{array}{l} \frac{1}{L^{bs}},f(i,j)\in T^{bs}\\ 0,\;\;\;\;\;\;otherwise \end{array}\right.$$

where *e* is the weight size of the path *T^bs^*, Δ*τ^bs^ij*(*t*) is the pheromone added at moment *t* of the shortest route, *T^bs^* is the shortest route, and *L^bs^* is the length of *T^bs^*.

**Figure 6:**
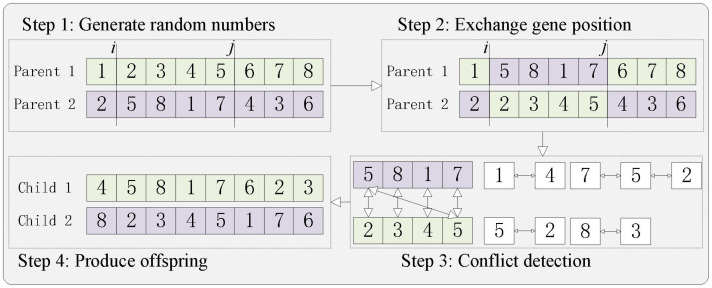
Schematic diagram of the crossover approach.

(4) Fitness. The fitness of the lowest comprehensive scheduling cost as the optimization objective, from the objective function of Equation 1 can be obtained from fitness, as shown in Equation 19:


(19) f=1/min y

(5) Selection, crossover. A roulette selection operator was used in the operator operation to randomly select and retain the optimal individuals based on the fitness ratio. The crossover method is PMX crossover, as shown in **Figure 6**. The detailed steps are as follows. Step 1: Randomly select the start and end positions of genes within the parent chromosomes (the selected positions on the two chromosomes are identical). Step 2: Exchange the positions of the two gene groups. Step 3: Perform conflict detection. Based on the swapped gene groups, establish a mapping relationship; taking the 7–5–2 mapping as an example, it is evident that in the results of Step 2, parent 1 has two instances of gene 7. These are then converted into gene 2 through the mapping relationship, and so forth until all conflicts are resolved. Step 4: Ultimately, the offspring are generated.

The specific steps for PMX are as follows **Algorithm 2**.

**Algorithm 2:** Crossover steps.

**Input**: Parent_1 and Parent_2% Paternal chromosome; **Output**: Child_1 and Child_2% Child chromosome; 1: *i*=*i*∈(1,chromosome_length);% Generate random numbers *i* 2: *j*=*j*∈(1,chromosome_length);% Generate random numbers *j* 3: **while** *i*==*j* **do** 4: *j*= *j*∈(1,chromosome_length);% Generate random numbers *j* 5: **end while** 6: **if** *i*<*j* **then** % Compare the size 7: *C*=[*i*,*j*]; 8: **else** 9: *C*=[*j*,*i*]; 10: **end if** 11: Segment_1=Parent_1(*C*) and Segment_2=Parent_2(*C*) 12: Parent_1(*C*)=Segment_2% Switch the location of two sets of genes 13: Parent_2(*C*)=Segment_1% Switch the location of two sets of genes 14: **While** Duplicate numbers exist in Parent_1 **do** % Conflict detection 15: Establish mapping relationship 16: **end while** 17: Child_1=Parent_1 18: **While** Duplicate numbers exist in Parent_2 **do** % Conflict detection 19: Establish mapping relationship 20: **end while** 21: Child_2=Parent_2

**Figure 7:**
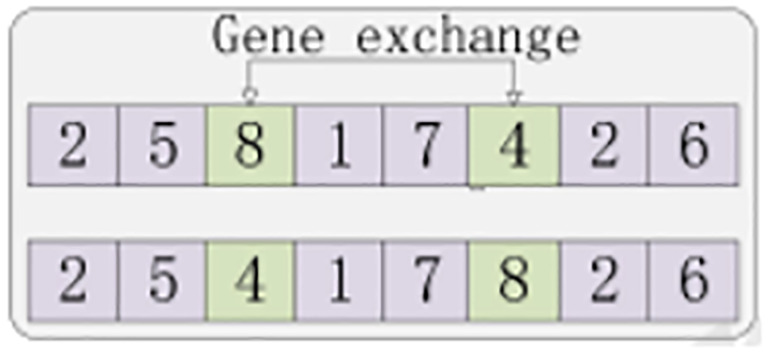
Schematic diagram of the variation.

(6) Mutation. Mutation improves the diversity of the population, and it is important to note that the choice of the mutation rate is a critical parameter. An inadequate mutation rate can lead the algorithm to get stuck in local optimal solutions, whereas an excessively high mutation rate may result in the algorithm losing its search direction. Therefore, the selection of the mutation rate should be fine-tuned based on the specific circumstances. The mutation steps are illustrated in **Figure 7**.

The specific steps of the mutation are as follows Algorithm 3.

**Algorithm 3:** Steps of mutation.

**Input**: chromosome **Output**: New_chromosome 1: *i*=*i*∈(1,chromosome_length);% Generate random numbers *i* 2: *j*=*j*∈(1,chromosome_length);% Generate random numbers *j* 3: **while *i*==*j* do** 4: *j*= *j*∈(1,chromosome_length);% Generate random numbers *j* 5: **end while** 6: *t1*=chromosome(*i*); 7: *t2*=chromosome(*j*); 8: chromosome(*i*)=*t2*; 9: chromosome(*j*)=*t1*; 10: New_chromosome=chromosome;

(7) Elite retention. Elitism is implemented to prevent population degradation. In this process, the best individual from each generation is duplicated. One of these top individuals is directly passed on to the next generation without any genetic alterations, while the other continues to participate in the evolutionary process as usual. The specific steps of Elite retention are as follows Algorithm 4.

**Algorithm 4:** Steps of Elite retention.

**Input**: elite **Output**: new_population 1: %Select the best individuals from the current population elite ← get_best_individual(current_population) 2: %Duplicate the best individuals elite_copy ← copy_individual(elite) 3: %Add the duplicated best individuals, elite_copy, to the offspring generated by crossover and mutation offspring ← crossover_and_mutate(selected_parents) offspring ← offspring ∪ {elite_copy} 4: % Directly add the original best individuals, elite, to the next generation population current_population ← {elite} ∪ select_from(offspring, population_size - 1) 5: %Update the population to the new generation new_population ← current_population

## 4 Example validation

### 4.1 Experimental data

**Figure 8:**
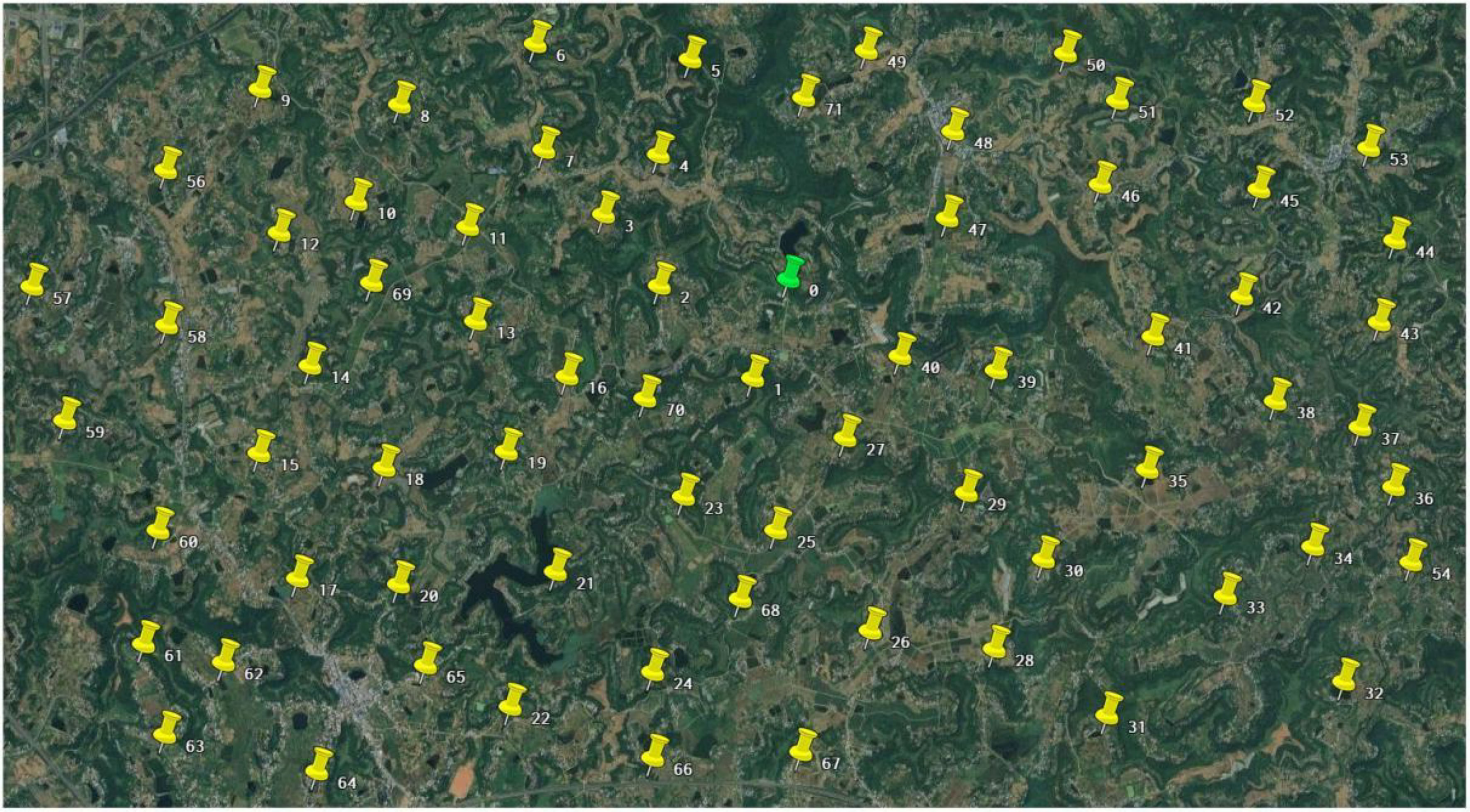
Map of the experiment.

To verify the effectiveness and advantages of the improved genetic algorithm proposed in this study for the emergency scheduling problem in case of harvester breakdown scenarios, the farmland data of this study were taken from Chuanwan District, Deyang City, Sichuan Province, as shown in **Figure 8**. Simulated operational tasks were set up based on the actual operational environment to verify the performance and stability of the algorithm. The algorithm was run on AMD Ryzen 7 5800H with Radeon Graphics 3.20 GHz, 16 GB of RAM, Windows 11, and MATLAB. The basic information about the management center of the agricultural machinery cooperative and the farmland is shown in **Table 2**, which mainly includes geographic location information, farmland area, and operation time window, where O denotes the management center of the agricultural machinery cooperative. The harvester was selected as the Lovol Gushen GE80S(4LZ-8E2), and its working efficiency in farmland is 1 hm^2^/h, and the traveling speed during the transfer of harvester is 24 km/h.

**Table 2:** Experimental data information.

No.	Longitude (°)	Latitude (°)	Area (Mu)	Time window (h)	No.	Longitude (°)	Latitude (°)	Area (Mu)	Time window (h)
0	104.77427378	30.96110084	–	–	36	104.82819557	30.94522465	8.99	10:40–12:00
1	104.77103233	30.95354261	7.54	08:00–09:00	37	104.82519150	30.94978858	7.79	10:20–12:20
2	104.76270676	30.96060863	10.18	08:00–09:00	38	104.81763840	30.95177602	7.71	10:20–11:20
3	104.75777149	30.96605499	6.29	12:50–16:00	39	104.79274750	30.95413147	7.48	08:00–09:40
4	104.76262093	30.97061793	8.76	13:30–16:10	40	104.78416443	30.95519876	8.59	08:00–10:20
5	104.76545334	30.97786665	7.94	14:00–18:50	41	104.80673790	30.95670766	7.27	09:00–10:10
6	104.75163460	30.97904406	10.12	11:30–13:00	42	104.81463432	30.95976221	7.99	09:30–11:00
7	104.75244999	30.97098590	8.87	12:00–13:20	43	104.82690811	30.95781173	7.61	11:10–16:30
8	104.73957539	30.97444473	9.36	11:00–12:00	44	104.82828140	30.96406784	8.55	11:10–17:20
9	104.72708702	30.97562218	9.79	12:20–16:00	45	104.81622219	30.96793171	7.24	13:30–17:30
10	104.73567009	30.96701176	8.24	10:00–11:20	46	104.80201721	30.96829969	7.18	13:10–17:40
11	104.74566936	30.96506142	6.02	16:00–18:00	47	104.78837013	30.96572381	8.94	12:20–18:00
12	104.72880363	30.96465663	8.62	12:00–17:00	48	104.78892803	30.97234737	8.41	12:00–18:00
13	104.74635601	30.95788533	7.58	09:00–10:20	49	104.78120327	30.97852895	9.16	13:20–18:00
14	104.73155022	30.95449950	6.73	11:00–14:20	50	104.79892731	30.97834498	8.62	11:00–18:00
15	104.72700119	30.94780109	7.65	10:40–14:20	51	104.80360508	30.97470230	7.58	10:20–18:00
16	104.75450993	30.95365302	9.88	08:00–10:00	52	104.81575012	30.97451833	6.73	10:20–17:40
17	104.73043442	30.93823111	6.99	13:00–16:30	53	104.82600689	30.97113308	7.65	10:20–17:00
18	104.73815918	30.94669691	7.79	14:00–17:40	54	104.82974052	30.93948262	7.88	10:20–17:20
19	104.74905968	30.94794831	9.71	12:00–17:00	55	104.77626801	30.92873381	6.99	10:20–17:10
20	104.73944664	30.93778940	7.48	13:00–16:50	56	104.71867561	30.96944042	7.58	10:20–17:50
21	104.75343704	30.93874644	8.59	08:00–09:50	57	104.70674515	30.96053503	6.73	10:20–17:20
22	104.74940300	30.92843930	7.27	08:00–12:00	58	104.71876144	30.95759091	7.65	10:20–17:40
23	104.76489544	30.94448851	9.99	08:00–10:00	59	104.70966339	30.95030384	9.88	10:20–17:40
24	104.76210594	30.93108981	7.61	09:30–11:20	60	104.71803188	30.94187518	9.99	10:20–11:40
25	104.77309227	30.94191198	8.55	08:00–09:30	61	104.71670151	30.93322490	7.79	10:00–11:20
26	104.78150368	30.93425561	7.24	14:00–17:50	62	104.72382545	30.93182606	9.71	12:00–17:00
27	104.77927208	30.94897886	7.18	12:00–18:00	63	104.71858978	30.92630411	7.48	09:00–10:30
28	104.79257584	30.93285678	8.94	13:00–17:30	64	104.73215103	30.92350620	8.59	11:10–17:00
29	104.79012966	30.94474616	8.41	08:00–10:00	65	104.74192500	30.93159598	7.27	11:10–17:00
30	104.79699612	30.93966667	9.16	08:00–10:30	66	104.76210594	30.92453702	6.99	10:00–11:20
31	104.80261803	30.92773985	8.62	12:20–17:00	67	104.77532387	30.92497880	7.61	10:20–17:20
32	104.82373238	30.93035357	7.58	11:20–17:00	68	104.76991653	30.93668510	8.55	09:00–11:00
33	104.81317520	30.93686915	6.73	09:30–11:20	69	104.73708630	30.96082943	7.24	10:00–11:30
34	104.82098579	30.94062370	7.65	10:20–11:20	70	104.76146221	30.95196005	7.18	11:40–18:00
35	104.80622292	30.94651288	7.88	09:00–10:30	71	104.77558136	30.97495987	9.94	10:20–17:20

### 4.2 Initial scheduling program

In the initial scheduling stage, the initial values are set for the algorithm parameters: The population size is 500, the maximum number of iterations is 1,000, the crossover probability is 0.9, and the variance probability is 0.05. The number of ants is 50, the pheromone importance factor *α* = 1, the heuristic factor *β* = 2, and the pheromone evaporation coefficient is 0.75. The harvester transfer cost is 10, the operation cost is 3, and the penalty cost is 0.5. The experimental data and algorithm parameters are incorporated into the objective function of the initial scheduling, the simulation solution of the algorithm is completed using MATLAB programming, and the results of the initial scheduling are shown in **Table 3**.

**Table 3:** Initial scheduling results.

Harvester no.	Operating route	Initial movement of costs (CNY)			Optimum total cost (CNY)
		Distance	Operating	Penalty	
*M*_1_	29>30>35>33>34>36>54>32>31>28>67>55>26>27	179.79	332.58	0	512.37
*M*_2_	1>25>68>24>66>22>65>64>62>17>20>18>19>70	155.15	333.69	0	488.84
*M*_3_	2>6>13>69>10>8>6>7>3>4>5>71>49>47	168.76	368.22	0	536.98
*M*_4_	40>39>41>42>38>37>43>44>53>52>45>46>51>50>48	155.51	349.20	0	504.71
*M*_5_	23>21>63>61>60>15>14>58>59>57>56>9>12>11	221.29	342.75	0	564.76

**Figure 9:**
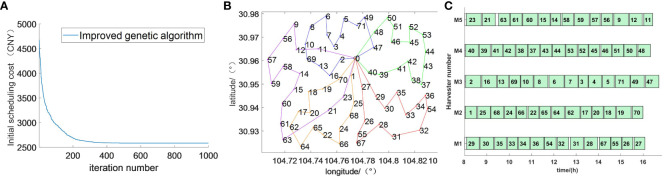
Initial scheduling scheme result graph. **(A)** Initial scheduling convergence graph. **(B)** Initial scheduling route map. **(C)** Initial scheduling Gantt chart.

The scheduling results of the initial scheme are shown in **Figure 9**. **Figure 9A** shows the convergence of the improved genetic algorithm in the operation process; by combining the advantages of the genetic algorithm and the ant colony algorithm, it can be concluded that the objective function tends to be stable in the generation of 470, which plays a positive role in improving the convergence speed and the solution accuracy. **Figure 9B** shows the route map of the initial scheduling scheme, from which it can be concluded that the farmland where each harvester works maintains the optimal distance. **Figure 9C** shows the Gantt chart of the initial scheduling scheme, which shows the sequence of the farmland worked by each harvester. The completion time of each harvester’s operation task is approximately the same, where the white part is the harvester’s transfer phase and the green part is the harvester’s operation phase.

### 4.3 Emergency scheduling program

In the contingency scheduling scheme, the improved genetic algorithm, the genetic algorithm, and the ant colony algorithm were used to compare different harvesters and different time periods when they break down. The contingency scheduling results were obtained by the three algorithms by performing operations 10 times at randomly set breakdown moments, and the optimal cost, average cost, standard deviation, and each scheduling cost obtained are shown in **Table 4**. As can be seen from **Table 4**, the optimal scheduling integrated costs using the improved genetic algorithm are 47.49% and 34.70% lower than the genetic algorithm and the ACO algorithm, respectively, after the first agriculture machinery breaks down at moment *t*1. After the second agriculture machinery breaks down at moment *t*2, the optimal scheduling integrated costs are reduced by 19.60% and 14.80%, respectively. After the third agriculture machinery breaks down at moment *t*3, the optimal scheduling integrated costs are reduced by 32.45% and 24.40%, respectively. In addition, the standard deviations of the improved genetic algorithm are smaller than those of the other two algorithms, which indicates that the improved genetic algorithm has a greater advantage in the accuracy of the optimal solution and can find the optimal solution in different harvesters and different time periods. Therefore, the improved genetic algorithm can meet the task requirements of emergency scheduling in case of a harvester breakdown.

**Table 4:** Emergency scheduling results.

Algorithm	Breakdown time	Broken down harvester	Various costs (CNY)				Optimum total cost (CNY)	Average cost (CNY)	Standard deviation
			Distance	Operating	Penalty	Offset cost			
Improved genetic algorithms	Time *t*1	*M*_1_	1,314.11	1,726.44	45.21	2,712.75	5,798.51	5,804.71	3.63
	Time *t*2	*M*_2_	944.79	1,726.44	0.00	513.29	3,184.52	3,193.52	5.74
	Time *t*3	*M*_3_	958.73	1,726.44	6.67	995.94	3,687.78	3,692.98	2.79
Genetic algorithm	Time *t*1	*M*_1_	2,410.02	1,726.44	300.22	6,605.95	11,042.63	11,052.63	6.31
	Time *t*2	*M*_2_	1,306.54	1,726.44	0.00	927.82	3,960.8	3,970.3	7.63
	Time *t*3	*M*_3_	1,719.40	1,726.44	15.01	1,998.55	5,459.4	5,472.9	8.62
Ant colony algorithm	Time *t*1	*M*_1_	1,431.03	1,726.44	121.36	5,600.34	8,879.17	8,890.87	7.66
	Time *t*2	*M*_2_	1,091.41	1,726.44	0.00	919.88	3,737.73	3,749.43	7.85
	Time *t*3	*M*_3_	1,231.07	1,726.44	16.54	1,904.44	4,878.49	4,891.49	8.66

**Figure 10:**
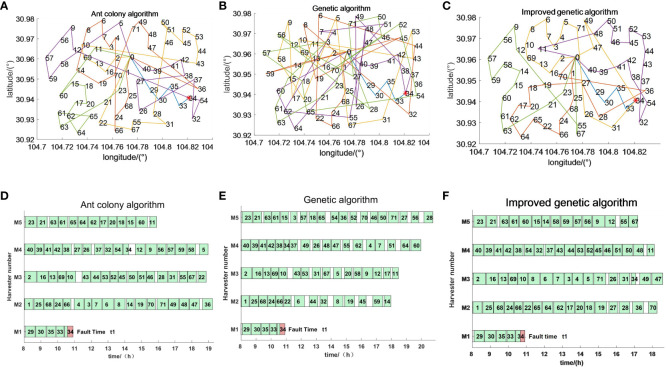
Emergency scheduling results at moment *t*1. **(A–C)** Emergency scheduling route map. **(D–F)** Emergency scheduling Gantt chart.

**Figure 11:**
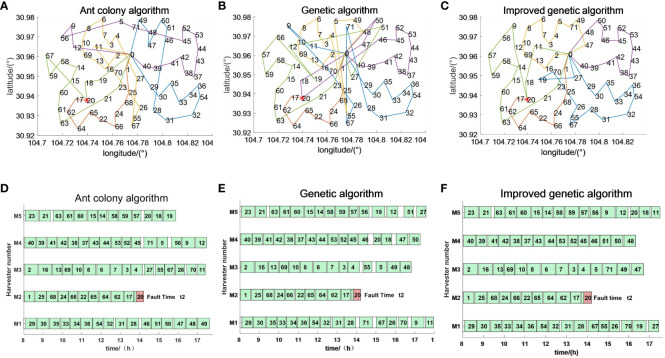
Emergency scheduling results at moment *t*2. **(A–C)** Emergency scheduling route map. **(D–F)** Emergency scheduling Gantt chart.

**Figure 12:**
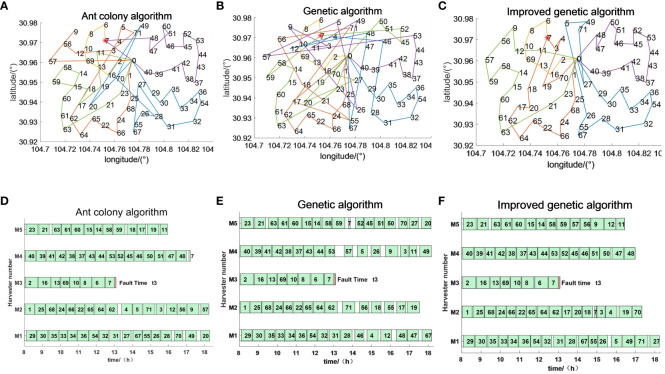
Emergency scheduling results at moment *t*3. **(A–C)** Emergency scheduling route map. **(D–F)** Emergency scheduling Gantt chart.

The emergency scheduling route map and Gantt chart for each fault stage are shown in **Figures 10**–**12**, where the red markers in the route map and Gantt chart are the locations where the harvester breaks down. From the route map, it can be intuitively seen that although all three algorithms are capable of completing the operation tasks in all farmlands, the operation route of the genetic algorithm is more complicated, and such an operation route increases the transfer time and prolongs the operation progress. The ant colony algorithm has a better operation route relative to the genetic algorithm, but the second half of the operation route of the farmland is beyond the scope of the time window relative to the improved genetic algorithm, which increases the penalty cost, whereas the improved genetic algorithm maintains the optimal distance and optimal cost in all three randomly set breakdown cases. From the operation progress Gantt chart, it can be concluded that the emergency scheduling scheme of the genetic algorithm and ant colony algorithm has the largest change from the initial scheduling scheme, while the emergency scheduling scheme of the improved genetic algorithm has less perturbation, and the maximum completion time is shorter than that of both the genetic algorithm and ant colony algorithm, so the improved genetic algorithm improves the efficiency of operation, has the optimal operation route and the shortest maximum completion time among all the algorithms, and is able to satisfy the harvester’s scheduling timeliness.

**Figure 13:**
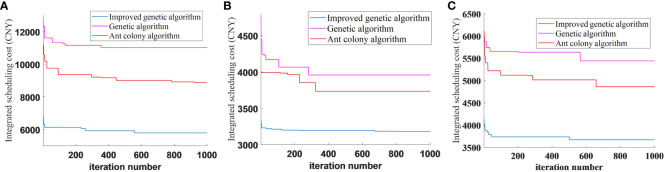
Convergence graph of contingency scheduling results. **(A)** Convergence graph at moment *t*1. **(B)** Convergence graph at moment *t*2. **(C)** Convergence graph at moment *t*3.

In addition to comparing the integrated scheduling costs at different time stages and under different harvester breakdown scenarios, the trends of the three algorithms during the iterative process were further analyzed. As shown in **Figure 13**, the genetic algorithm has a strong global search ability, but it is easy to fall into local optimal with the ant colony algorithm, while the improved genetic algorithm has the advantage of high convergence efficiency in three randomly set breakdown scenarios, which is due to the initial optimization search through the genetic algorithm to obtain the optimal solution quickly, and then the ant colony algorithm is used to carry out a secondary search for the sub-optimal solution and finally merge the populations to obtain the optimal solution. The improved genetic algorithm combines the advantages of the two algorithms; the first step of the algorithm, to a certain extent, determines the size of the optimal solution, and it can be seen from the figure that the improved genetic algorithm can greatly improve the efficiency of the algorithm’s initial search. More specifically, it can improve the evolutionary efficiency of the algorithm, optimize the overall size of the solution in fewer iterations, and reduce the search time. The improved optimization performance of the algorithm is evidenced not only by the high optimization accuracy but also by the highly stable results.

### 4.4 System design

**Figure 14:**
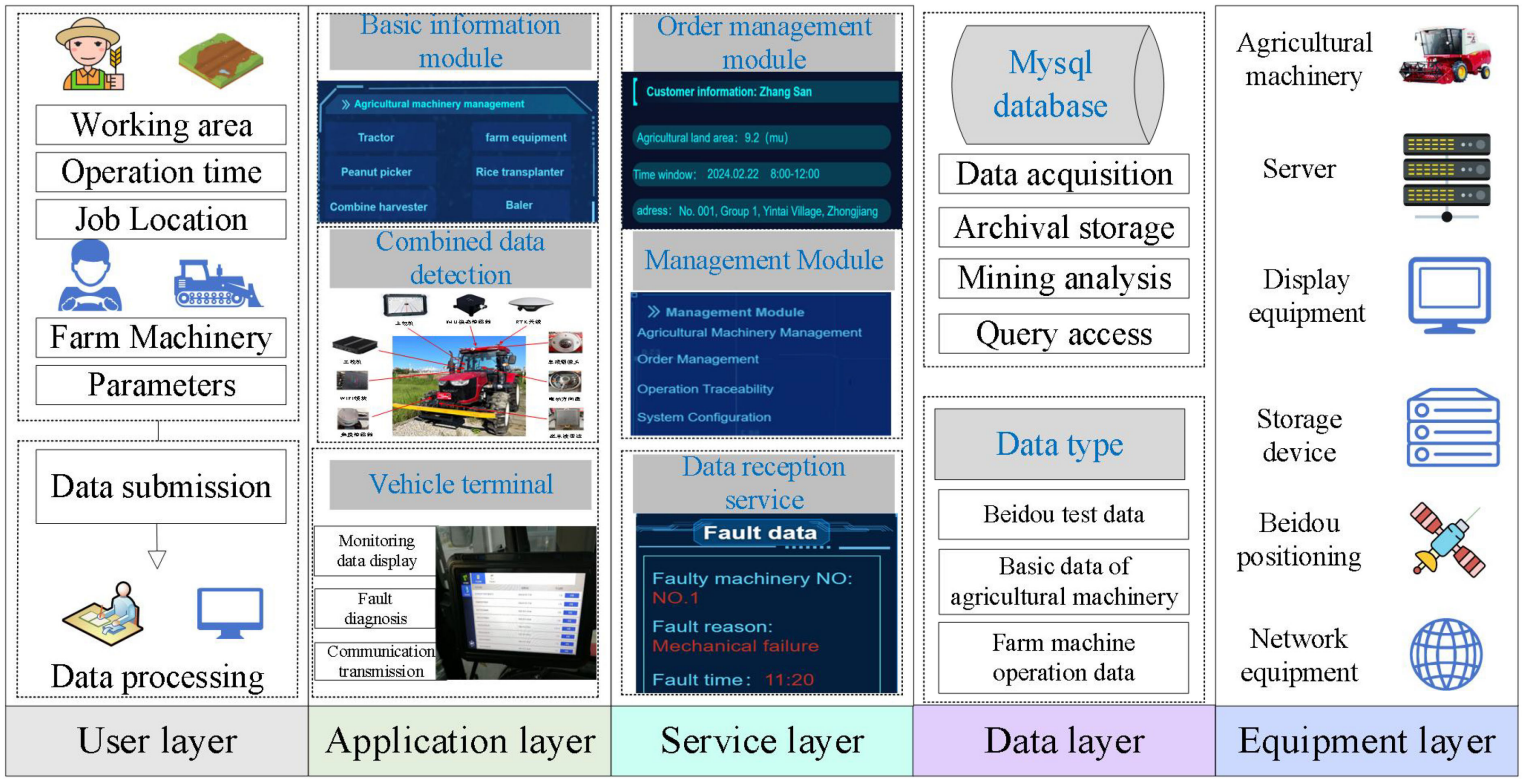
System framework diagram.

The study presents a scheduling model and algorithm for agricultural machinery, leading to the development of a visual management system. This system utilizes modern information technology and intelligent tools to create an information interaction platform for agricultural management departments, machinery cooperatives, farmers, and households. It enables precise analysis of agricultural machinery operations scheduling, real-time supervision, and handling of massive operational data. The system is structured into five layers: user, application, service, data, and equipment. The framework is illustrated in **Figure 14**.

**Figure 15:**
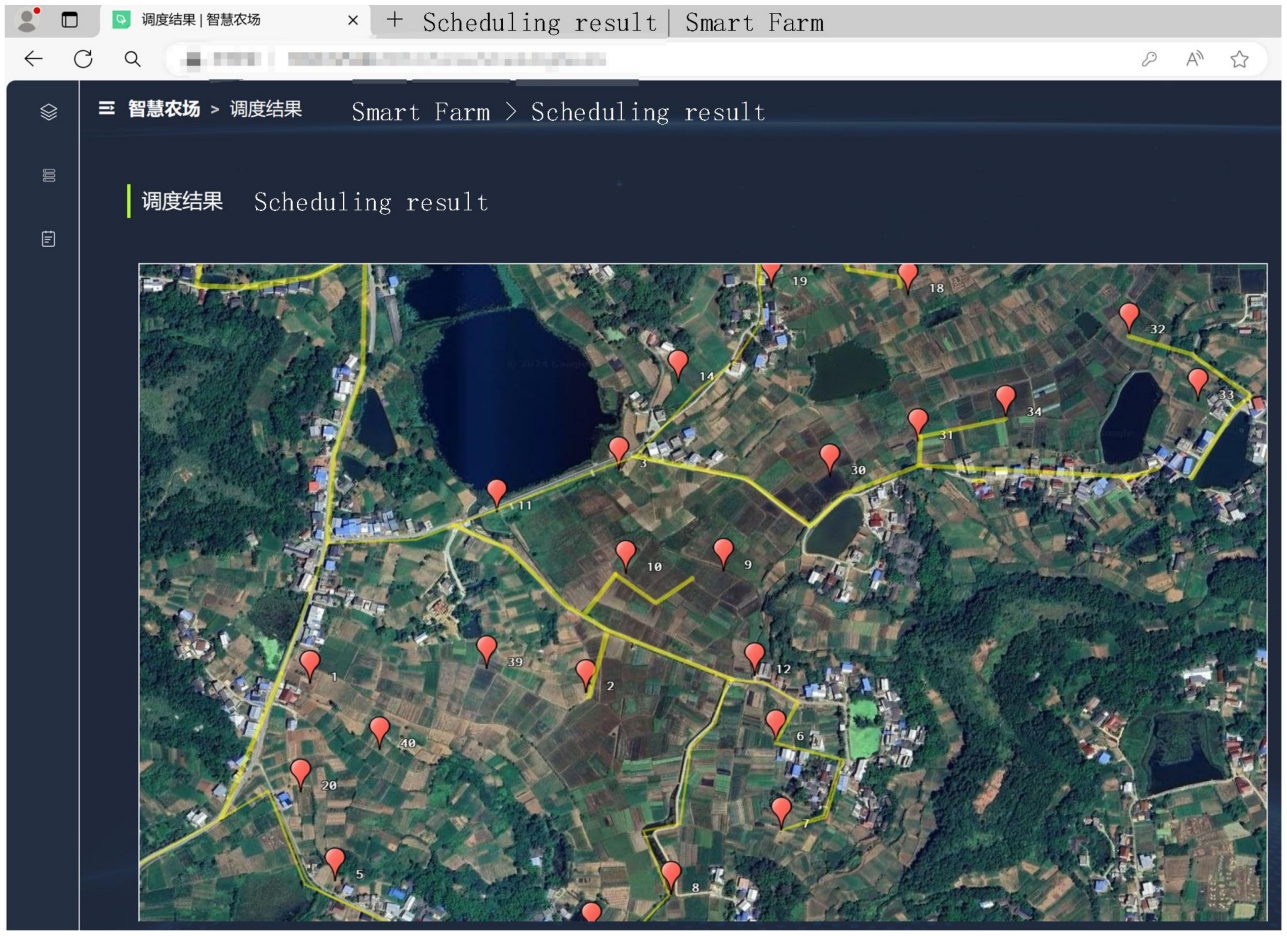
Breakdown scheduling visualization interface.

The system collects fault information and then uploads agriculture machinery data to the back-end of the system to form information interaction and achieve intelligent decision-making, and the visualization of scheduling is shown in **Figure 15**. The implementation of the system design can effectively achieve human–computer interaction and improve the overall management service level.

### 4.5 Discussion

**Table 5** compares recent studies on the scheduling of agricultural machinery, including scheduling objectives and research methods, and lists the characteristics of each method to better explain the experimental results obtained in this study.

**Table 5:** Comparison of agriculture machinery scheduling studies for various scheduling objectives.

No.	Reference	Objective	Method	Features
1	Ma et al. (2022)	Agricultural machinery dynamic scheduling	Multi-population cooperative co-evolutionary genetic algorithm	Agricultural machinery scheduling meeting dynamic demands, reasonably inserting dynamic orders into the initial scheduling plan.
2	Huang et al. (2023)	Multi-region agricultural machinery scheduling	Hybrid particle swarm optimization algorithm	Divide areas using a Voronoi diagram and assign farmlands to service centers within the region.
3	Li et al. (2023)	Harvester and grain transport vehicle coordination, dynamic scheduling	Hybrid optimization algorithm of NSGA-III and ant colony algorithm	Implemented dynamic time windows for grain transport vehicles and local dynamic obstacle avoidance routes, minimizing the transfer distance and number of grain transport vehicles.
4	Cao et al. (2021)	Multi-agricultural machinery cooperative scheduling	Improved ant colony algorithm	Resolved the “proximity” issue in task allocation, avoiding overloading of some agricultural machinery and idleness of others, thus shortening the operation cycle.
5	Seyyedhasani and Dvorak (2018)	Agricultural machinery dynamic scheduling	Dynamic routing algorithm	For three dynamic scenarios, scheduling within a single farmland is planned, providing an adaptive scheduling capacity to handle continuous changes.
6	Chen et al. (2023)	Crop protection, drone scheduling, dynamic scheduling	Levy simulated annealing algorithm	The proposed algorithm performs better in both static and dynamic planning scenarios. It offers more advantages in terms of drone adjustment distance and total operation time.
7	This study	Harvester malfunction scheduling, dynamic scheduling	Hybrid optimization algorithm of genetic algorithm and ant colony algorithm	Comparing the three algorithms, the improved genetic algorithm satisfies the dynamic scheduling of harvesters in case of malfunction, better ensuring the timeliness of scheduling.

NSGA-III, non-dominated sorting genetic algorithm III.

In this study, the proposed mathematical model and improved algorithm for the problem of the phenomenon of agricultural machines breaking down during the execution of scheduling tasks have the following benefits in terms of results. First, the initial scheduling problem is solved, and the managers of agricultural cooperatives can make quick decisions to allocate the agriculture machinery in the agriculture machinery pool to the operating farmland with high quality. Second, in the event that agricultural machinery breaks down during the scheduling task and is unable to regain operational capability in a short period of time, they are able to respond quickly to the remaining farmland and make timely emergency strategies. Finally, this research study can also be extended to the emergence of new operating farmland situations during the execution of scheduling tasks to develop better emergency scheduling strategies.

Issues related to this study should not be ignored. First, for scheduling agricultural machinery with loading capacity, its own capacity should also be considered when a breakdown occurs. Second, as the system contains many data, including job retrospective data, the data security and system maintenance of the management center should be strengthened to prevent data privacy leakage or even system crash.

## 5 Conclusion

We addressed an emergency scheduling problem where a harvester breaks down during the execution of a scheduled task and is unable to restore its operating capability in a short time. The objectives were to achieve the lowest comprehensive scheduling cost and resume and complete the remaining farm operations as soon as possible. Upon analysis of influencing factors in agricultural machinery operations, we established an emergency agricultural machinery scheduling model with the lowest comprehensive scheduling cost using an improved genetic algorithm that combined genetic and ant colony algorithms. Finally, compared with the scheduling solutions of the genetic algorithm and the ant colony algorithm, the scheme obtained in this study optimizes the sequence of agricultural machine operation and can meet the emergency scheduling task demands of the harvester in the event of a breakdown. The main conclusions of this study are as follows:

(1) Combined with the current situation of agricultural machine scheduling under a disturbance event, the emergency scheduling problem is proposed when an agricultural machine breaks down and can no longer restore its operating ability in a short time. Combining the influencing factors and characteristics of the scheduling process of agricultural machines, an objective function with the goal of minimizing the operating time offset is added to the initial scheduling. After that, the problem is solved by the improved genetic algorithm, and the results show that the optimal integrated scheduling cost of the improved genetic algorithm in the three breakdown stages is reduced by 47.49%, 19.60%, and 32.45% compared with the genetic algorithm’s 34.70%, 14.80%, and 24.40% compared with the ACO algorithm, which verifies the effectiveness of the improved genetic algorithm.

(2) To avoid getting trapped in local optima during the search for the global optimum, an improved algorithm combining GA and ACO was designed. The algorithm combines the advantages of the two algorithms; convergence speed is fast, and at the same time, it has a good global optimization ability; through three different time periods and different breakdowns of agricultural machinery samples, the three algorithms are compared, and the results show that the improved genetic algorithm enhanced convergence efficiency, resulting from the combination of a genetic algorithm and an ant colony algorithm.

(3) This study designed and developed a set of agricultural machinery scheduling visualization management systems that provide intelligent information references for agricultural machinery scheduling operation scenarios. It can effectively provide all-around service information interaction, realize multi-position integration and complete functions, meet the needs of agricultural machinery operation service scheduling operations, and provide support for the reasonable planning of the whole scheduling scene.

## Data availability statement

The original contributions presented in the study are included in the article/supplementary material. Further inquiries can be directed to the corresponding author.

## Author contributions

HL: Conceptualization, Data curation, Formal analysis, Funding acquisition, Investigation, Methodology, Project administration, Resources, Software, Supervision, Validation, Visualization, Writing – original draft, Writing – review & editing. LZ: Conceptualization, Data curation, Investigation, Methodology, Software, Validation, Visualization, Writing – original draft, Writing – review & editing. BZ: Conceptualization, Data curation, Investigation, Methodology, Software, Writing – review & editing. JT: Conceptualization, Data curation, Methodology, Software, Writing – review & editing. JL: Data curation, Investigation, Methodology, Software, Validation, Writing – review & editing. SW: Writing – review & editing, Formal analysis, Funding acquisition, Project administration, Resources, Supervision.
